# Environmental Pollution as a Risk Factor in Testicular Tumour Development: Focus on the Interaction between Bisphenol A and the Associated Immune Response

**DOI:** 10.3390/ijerph16214113

**Published:** 2019-10-25

**Authors:** Karen Elizabeth Nava-Castro, Ricardo Ramírez-Nieto, Lucía Angélica Méndez-García, Manuel Iván Girón-Pérez, Mariana Segovia-Mendoza, Migdalia Sarahy Navidad-Murrieta, Jorge Morales Montor

**Affiliations:** 1 Laboratorio de Genotoxicología y Mutagénesis Ambiental, Departamento de Ciencias Ambientales, Centro de Ciencias de la Atmósfera, Universidad Nacional Autónoma de México, Ciudad de México 04510, Mexico; karlenc@atmosfera.unam.mx; 2 Departamento de Inmunología, Instituto de Investigaciones Biomédicas, Universidad Nacional Autónoma de México, AP 70228, Ciudad de México 04510, Mexico; psycardirko@hotmail.com (R.R.-N.); monaco445@yahoo.com.mx (M.S.-M.); 3 Unidad de Investigación en Medicina Experimental, Facultad de Medicina, Universidad Nacional Autónoma de México, Hospital General de México Dr. Eduardo Liceaga, Ciudad de México 06726, Mexico; angelica.mendez.86@hotmail.com; 4 Secretaría de Investigación y Posgrado, Laboratorio de Inmunotoxicología, Universidad Autónoma de Nayarit, Cd de la Cultura s/n, Tepic 63155, Nayarit, Mexico; ivan_giron@hotmail.com (M.I.G.-P.); ivan_giron@uan.edu.mx (M.S.N.-M.); 5 Centro Nayarita de Innovación y Transferencia de Tecnología, Unidad Especializada Laboratorio Nacional para la Investigación en Inocuidad Alimentaria (LANIIA-Unidad Nayarit), Calle tres s/n, Tepic 63173, Nayarit, Mexico

**Keywords:** environmental pollution, BPA, endocrine disruptors, cancer, tumour, testicular tumours

## Abstract

Bisphenol A (BPA) is an endocrine disruptor to which animals and humans are highly exposed. Many reports have established a relationship between BPA exposure and breast cancer incidence, especially during critical periods of development. However, its effects on the immune response in testicular tumour growth have not yet been described. Thus, we wanted to analyse the effect of perinatal BPA exposure in pregnant female mice and the immune response modulation and tumour growth in an intratesticular cancer model in offspring male mice. Pregnant female mice were exposed to a dose of 250 mg/kg/day/body weight of BPA in their drinking water. In adulthood, male offspring underwent intrascrotal inoculation with 4T1 cancer cells. On day 21 after inoculation, mice were euthanised, and serum was obtained to measure BPA levels using HPLC coupled to mass spectrometry. The percentages of immune cell populations in peripheral lymph nodes (PLN), the spleen and tumours were evaluated by flow cytometry. In addition, the tumour expression of IL-10, TNF-α and TGF-β was analysed by RT-PCR. Of note, we found detectable circulating levels of BPA in the offspring of mothers exposed to it while pregnant. Remarkably, BPA treatment promoted tumour growth by about 75% compared to mice coming from female mice that did not receive the compound. Perinatal exposure to BPA modulated the percentages of different immune cells in the spleen and PLN. In addition, the expression of inflammatory-related cytokines (IL-10 and TNF-α) in the tumours was significantly enhanced compared to control and vehicle groups. In conclusion, the perinatal BPA administration in pregnant female mice modulated different cellular and molecular immune components that resulted in outstanding testicular tumour size in male offspring.

## 1. Introduction

During the past 70 years, the presence of human-made chemical contaminants in the environment has quickly increased. Environmental pollutants have harmful effects on human and animal health. Most of these compounds are considered as endocrine-disrupting compounds (EDCs), which can modify the synthesis, secretion, metabolism, transport and elimination of some endogenous steroid hormones, and consequently, they cause adverse health effects in the organism or its progeny [1].

One of the most commonly used compounds in the plastics manufacturing industry is bisphenol A (BPA) (2,2-(4,4-dihydroxydiphenyl) propane), which is used as a monomer for the production of polycarbonate plastic products, epoxy resins, flame-retardants, food containers, baby bottles, compact discs, adhesives, optical lenses, thermal paper, powder paints and dental resins, among other products. BPA can leach from these and other products through high temperatures or acidic or basic changes, and as a result, routine ingestion of BPA by different organisms is presumed [2,3].

Exposure to nanomolar concentrations of BPA from the environment is ubiquitous and continuous via different routes. In experimental animal models, BPA has been shown to affect the brain, liver, gut, adipose tissue, pancreas, mammary gland and reproductive tract of exposed animals. In humans, similar concentration ranges have impacted malignant breast, prostate, male germ or adipocyte cell lines [4,5,6,7]. During the perinatal stage, a process called hormonal "imprinting" takes place, which is defined as a period in which natural hormones first encounter their receptors. Thus, exposure to endocrine disruptors can alter physiological processes throughout life. Furthermore, several studies have shown that endocrine disruptors not only affect the endocrine system, but also interact with the immune system, as immune cells also respond to hormone effects and express hormone receptors [8]. In this sense, BPA exposure during early developmental stages may alter the imprinting process, affecting the production of antibodies, cytokines, and in general, the proliferation of different immune cells during adult life [9]. Few reports have focused on the study of the effects of BPA on the immune system. For instance, BPA can influence the activation of T helper cells, favouring TH1 or TH2 polarisation [10,11,12]. In line with that, our laboratory demonstrated that only a single neonatal dose of BPA in mice can also alter the immune response itself, promoting tumour development into adulthood with a concomitant increment of regulatory T lymphocytes (Tregs) infiltrating into the tumour. These effects were accompanied by the decreased expression of TNF-α and IFN-γ, as well as the M2 macrophage marker Fizz-1 in the BPA-exposed group [13]. The above suggests that BPA exposure in early life can contribute to breast cancer development and progression through the modulation of the antitumoural immune response.

In contrast, testicular cancer is the most common solid malignancy affecting males aged from 15 to 35 years old, accounting for about 1% of all cancers diagnosed in men [14]. Germ cell tumours (GCTs) represent 95% of testicular cancers, and two main types are pure-seminoma germ cell tumours and non-seminoma germ cell tumours [15]. Studies have suggested that foetal exposure to environmental EDCs, with oestrogenic effects, could participate in testicular germ cell carcinogenesis, influencing the fate of germ stem cells that share molecular markers with the malignant germ cells. In vitro, it has been shown that low doses of BPA promote seminoma cell proliferation through the G-protein-coupled oestrogen receptor (GPER), a membrane G-protein-coupled receptor (GPCR) [16]. In vivo, neonatal exposure to BPA in male mice modifies the prostate gland development, increasing the susceptibility to cancer generation through activation of epigenetic mechanisms [17]. However, now work related to the effect of BPA on the immune response in male testicular cancer has been performed. Of note, no experimental model to study testicular cancer has not been developed before.

In this work, we developed a testicular cancer model in which offspring males of BPA-exposed pregnant females underwent an intratesticular inoculation with 4T1 cancer cells; the size and weight of testicular tumours were studied and related with both intra-tumoural and systemic immunity. To our knowledge, this is the first integrative work where testicular tumours were induced in BALB/c mice, and the effect of BPA on the immune response against intratesticular tumour growth was studied.

## 2. Materials and Methods

### 2.1. Ethics Statement

Animal care and experimental practices were conducted at the Unidad de Modelos Biológicos (UMB) of the Instituto de Investigaciones Biomédicas (IIB), Universidad Nacional Autónoma de México. All experimental procedures in the animals were approved by the Institutional Care and Animal Use Committee (CICUAL), (Authorisation number 155), according to the Mexican regulation (NOM-062-ZOO-1999), and the Guide for the Care and Use of Laboratory Animals Recommendations [National Institute of Health (NIH) of the United States of America]. Euthanasia of experimental animals was performed humanely using 5% sevoflurane (Abbot, México City, México) before cervical dislocation.

### 2.2. Animals

Syngeneic strain BALB/c AnN (H2-d) mice were purchased from Harlan México (Facultad de Química, UNAM, México). The animals were housed at UMB with controlled temperature (22 °C) and 12-h light-dark cycles, with water and Purina LabDiet 5015 (Purina, St. Louis, MO, USA) chow ad libitum. Females and males were placed in “concubinage” for a week and a half. The females were weighed on a digital weighing scale to monitor weight changes and verify the pregnancy status.

### 2.3. Preparation of BPA

BPA (purity 97% of CAS number 80-50-7) was obtained from Sigma-Aldrich (St Louis, MO, USA), and dissolved in pure corn oil (Sigma-Aldrich) at a concentration of 250 mg/kg/bw. For female administration, 50 mg of BPA was diluted in 50 µL of ethanol, and from this dilution, 50 µL was dissolved in 50 mL of water.

### 2.4. Perinatal BPA Exposure in Mice

BPA exposure was started once the pregnancy status of the females was confirmed (≈11th day of gestation). BPA was added to their drinking water in glass drinking fountains and placed for consumption by the animals until weaning. Water containers were changed every third day to administer a dose of 250 mg/body weight/day. The same amount of ethanol (25 µL) was added to the drinking water of the vehicle group, while control animals received just water.

### 2.5. Quantification of BPA Serum Levels

After serum was obtained from the animals, we performed a simple mass organic protocol extraction from serum. The BPA serum samples were reconstituted in 500 µL of HPLC grade methanol and analysed using the Acquity series H UPLC-MS/MS system (Waters, Milford, MA, USA) with a triple quadrupole mass spectrometer Xevo TQ-S (Waters, Milford, MA, USA). Each sample was automatically injected through a Sample Manager system—FTN Acquity from Waters (Waters, Milford, MA, USA). An Acquity UPLC BEH C_18_ 1.7 µm, 2.1 × 50 mm column was used.

### 2.6. Cell Culture

The 4T1 cell line (ATCC^®^ CRL-2539) was kindly donated by Dr. Pedro Ostoa-Saloma and cultivated in RPMI 1640 medium (Sigma, St. Louis, MO, USA), supplemented with 10% FBS (ByProductos, Guadalajara, México). Subculturing was carried on at 70 to 80% confluence. After the second subculture, cells were harvested and resuspended in 0.9% saline solution in a concentration of 250,000 cells/mL for tumour inoculation.

### 2.7. Tumour Induction Model

At sexual maturity (8 weeks old), mice from each exposure group were randomised into secondary experimental groups: the control (with and without tumour induction), vehicle (with and without tumour induction) and BPA (with without and tumour induction) groups. 1 × 10^3^ cells of the 4T1 cancer line were injected into the scrotum. Tumour growth was monitored for 25 days.

### 2.8. Flow Cytometry Analysis

The spleen, peripheral lymph nodes (PLN) and tumours were mechanically disaggregated using a 50 µm nylon mesh and washed with PBS. Spleen erythrocytes were lysed with ACK buffer (150 mM NH_4_Cl, 10 mM KHCO_3_, 0.1 mM Na_2_EDTA, pH 7.3) for 10 min and washed with PBS. Tumour samples were finely cut and incubated for 20 min in digestion medium [RPMI 1640, 10 U/mL DNase (Roche, Mannheim, Germany), 0.5 mg/mL type IV Collagenase (Sigma, St. Louis, MO, USA)]. Digestion was stopped by adding 50 µL of FBS and disaggregation was performed, followed by a PBS wash. The cells from all tissues were resuspended in FACS buffer (PBS, 2% FBS, 0.02% NaN_3_). Approximately 1 × 10^6^ cells were incubated with anti-CD16/CD23 (TruStain^®^, BioLegend, San Diego CA, USA) for 30 min at 4 °C, washed and stained. For the characterisation of cellular subpopulations, the following antibodies were used: APC Cy7-coupled anti-CD3ε (145-2C11), PE-coupled anti-CD4 (GK1.5), AlexaFluor^®^647-coupled anti-Foxp3 (150D), PerCP-coupled anti-CD8 (53-6.7), Alexa Fluor^®^ 647-coupled anti-F4/80 (BM8), PE-coupled anti-NKp46 (29A1.4) (all from BioLegend, San Diego, CA, USA) and VioletFluor^®^ 450-coupled anti-CD25 (PC61.5) (Tonbo biosciences, San Diego, CA, USA). For intranuclear staining, Foxp3/Transcription Factor Staining Buffer kit (Tonbo biosciences, San Diego, CA, USA) was used, according to the manufacturer’s instructions. For detection of the oestrogen alpha receptor, rabbit polyclonal anti-ERα (H-184) (Santa Cruz Biotechnology, Dallas, TX, USA) was used, followed by DyLight^®^ 488-coupled donkey anti-rabbit IgG (BioLegend, San Diego, CA, USA). Samples were collected using an Attune cytometer (Life Technologies, Waltham, MA, USA), with a blue and a red laser, and data were analysed using FlowJo software (Treestar Inc., Ashland, OR, USA).

### 2.9. Haematoxylin-Eosin Staining

The samples were placed in filter cassettes and post-frozen in 4% cold paraformaldehyde (pH 7.4) for 20 min. Tissues were included in Tissue-Tek. Tissues were frozen, and 6 µm thick sections were cut using a cryostat. Haematoxylin-eosin staining was performed through a series of consecutive steps where the samples were placed as follows: Xylol for 5 min; 100% alcohol 5 min; 95% alcohol 5 min; 70% alcohol for 5 min; washing with running water; Haematoxylin staining 10 min; alcohol/acid 5 min; washing with running water; Eosin staining 30 s; 70% alcohol 5 min; 95% alcohol 5 min, and 100% alcohol 5 min. Finally, a cover with acrylic resin was placed on top of the samples.

### 2.10. RT-PCR Assays

Tumour tissue samples were frozen in TRIzol^®^ reagent (Ambion, Carlsbad, CA, USA) immediately after collection. Total RNA was extracted with the same reagent, following the manufacturer’s protocol. Briefly, the tissue was disrupted in TRIzol^®^ reagent (1 mL/0.1 g tissue), and 0.2 mL of chloroform was added per each mL of reagent. After 15 min of centrifugation at 13,000 rpm, the aqueous phase was recovered. RNA was precipitated with isopropyl alcohol, washed with 75% ethanol and dissolved in RNAse-free water. The RNA concentration was measured by absorbance at 260 nm, and its integrity was verified by agarose gel electrophoresis (1.0%). Total RNA samples were reverse-transcribed, using M-MLV Reverse Transcriptase (Promega, Madison, WI, USA) and dT12–18 primer (Invitrogen, Carlsbad, CA, USA). cDNA was amplified by semi-quantitative PCR, using TaqDNA polymerase (Biotecnologías Universitarias, UNAM, México) and the *Mus musculus*-specific primers (Table 1). The relative expression percentage of each amplified gene was measured by optical density analysis (OD), using the 18S-ribosomal RNA as a constitutive control (Table 1).

### 2.11. Statistical Analysis

The general experimental design considered two independent variables: neonatal exposure (Control, Vehicle of BPA) and testicular tumour induction (Control, Vehicle of BPA). For the analysis of the tumour weight and tumour cell microenvironment data, only the exposure (BPA) variable was considered, as all animals belonged to the 4T1 group. Data from two to three independent experiments were graphed as the mean ± standard deviation and analysed with the Prism 6^®^ software (GraphPad Software Inc, La Jolla, CA, USA). The normality of data distribution was assessed with a Shapiro–Wilk test. Thereafter, a one-way ANOVA (α = 0.05) was performed, followed by a Tukey post-hoc test. Differences were considered significant with a *p*-value < 0.05. In data regarding oestrogen receptor expression, both independent variables were considered and therefore, a two-way ANOVA (α = 0.05) was performed, followed by a Holm–Šidák post hoc test, with the same significant difference criterion.

## 3. Results

### 3.1. BPA-Serum Levels

**Figure 1:**
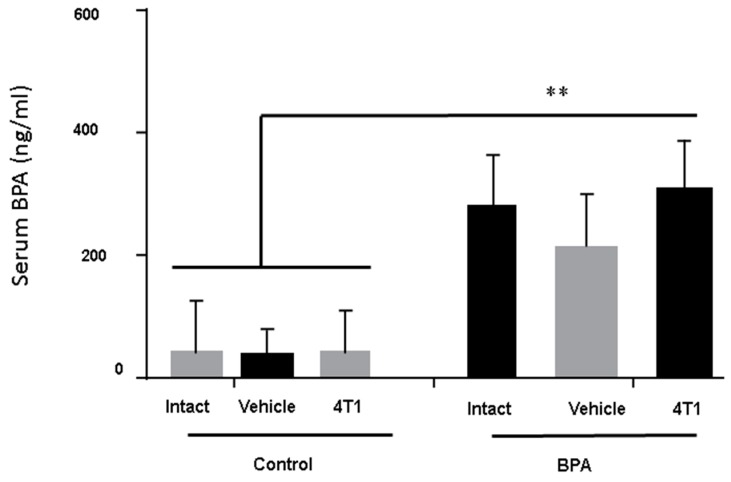
Bisphenol A (BPA) serum levels in male BALB/c mice. The BPA serum levels were measured in euthanised animals in control groups (without BPA treatment) *n* = 9, Vehicle (Ethanol) *n* = 7 and in the offspring of BPA-exposed females (BPA dose of 250 ng/kg/day) *n* = 7. Data are expressed as the mean and standard deviation of the tumour mass. ** *p* < 0.01.

Serum levels of BPA were measured in all the groups of euthanised animals. It was important to evaluate the BPA serum levels, so we could be sure that changes in immunity were due to BPA treatment. Figure 1 clearly shows that although all animals had baseline levels of this compound, only the sons of the mothers exposed to BPA had a significant increase of serum BPA levels around ten-fold compared to the control or vehicle groups.

### 3.2. Tumour Induction

**Figure 2:**
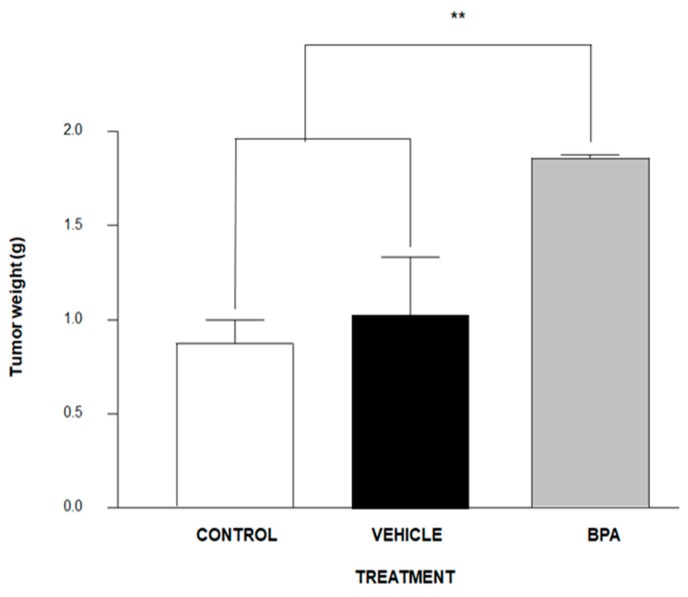
Tumour development after inoculation of 1 × 10^3^ 4T1 cells in the scrotum of male BALB/c mice. Control (no treatment) *n* = 9, Vehicle (Ethanol) *n* = 7 and offspring of BPA-exposed female (BPA 250 ng/kg/day) *n* = 7. Data are expressed as the mean and standard deviation of the tumour mass. ** *p* < 0.01.

BALB/c male mice of eight-week-old were inoculated with 1 × 10^3^ 4T1 cancer cells in the scrotum. Of note, we decided to inject this cell line into the scrotum because it comes from a tumor of BALBc mice, offering an alternative for the studying of testicular-like tumor models, since to date, there are no alternatives for the study in this type of tumors that are not xenotransplantation. As is shown in Figure 2, the tumour weight of the experimental group derived from pregnant female mice administered BPA increased significantly (≈75%) compared to the control vehicle group.

### 3.3. Haematoxilyn–Eosin (H&E) Staining

**Figure 3:**
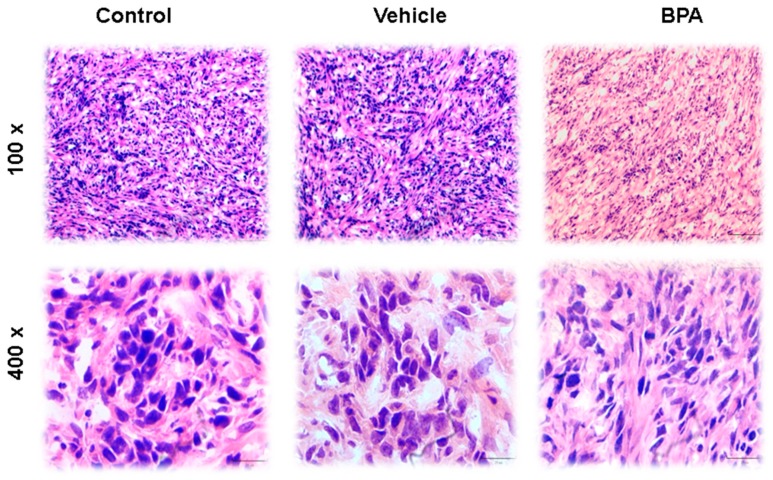
Histological sections of tumours stained with haematoxilyn–eosin (H&E). The images show specific characteristics of tumour cells, such as the presence of a disordered epithelium, necrotic areas and hyperchromatic nuclei in all groups—control, vehicle and BPA. In the images shown, the tumours have well-defined borders, round nuclei with fine chromatin, and rare nucleoli. All tumors featured papillary, solid, haemorrhagic, and sclerotic growth patterns at low magnification (100×). At higher magnification (400×) the merging of different growth patterns are shown. Images are displayed at 100× and 400× resolutions.

Tumor histology features four distinct architectural patterns and two neoplastic cell types. The tumors have papillary, sclerotic and solid, with haemorrhagic growth patterns. As shown in Figure 3 (100× and 400×), the papillary pattern is the most plentiful but may merge with sclerotic areas because the foci of hyaline collagen can expand papillary stalks as well as form solid sheets. The solid pattern may contain small tubules, and the haemorrhagic areas feature large, blood-filled spaces lined by tumor cells. Cuboidal tumor cells—also called surface cells—line papillae and cysts and form tubules, whereas round cells—so-called stromal cells—fill the papillary cores and form the sheets in solid areas. The surface cells are cuboidal with voluminous eosinophilic cytoplasm and prominent nuclei. Intranuclear inclusions and multinucleation are often observed (Figure 3). The stromal cells are slightly smaller than the surface cells and are typically described as having well-defined borders, clear to eosinophilic cytoplasm, centrally located round to oval nuclei with fine, dispersed chromatin, and usually indiscernible nucleoli. Cytoplasmic vacuoles may even resemble signet-ring cells. Focal nuclear atypia can be seen in either cell type, and mitotic indices are low, with reports of no more than one per ten high-power (400×) fields. Associated histologic findings include xanthoma cells, cholesterol clefts, haemosiderin, and calcification, as well as laminated whorls, granulomas, necrosis, mature adipose tissue, and numerous mast cells. Nests of neuroendocrine cells can also be seen, and on very rare occasions, sclerosing haemangioma may be combined with a typical carcinoid tumor. Different images of the scrotal-4T1 tumour model are shown in Figure 3. Interestingly, BPA did not induce any dramatic changes in tumour cell morphology in scrotal tumours of the offspring male mice of female mice exposed to this compound.

### 3.4. Percentage of Immune Cell Populations

**Figure 4:**
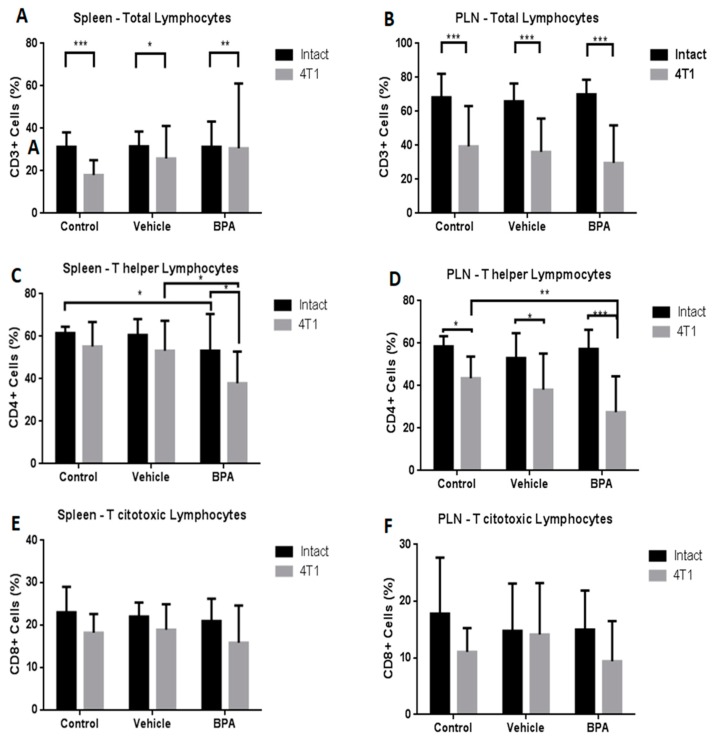
Percentages of T cell subpopulations in the spleen and peripheral lymph nodes (PLN) in the offspring of BPA treated mice. Total lymphocytes (CD3+) (**A**,**B**), T helper cells (CD4^+^) (**C**,**D**) and T cytotoxic cells (CD8^+^) (**E**,**F**) in control (*n* = 7), vehicle (*n* = 6), vehicle/4T1 (*n* = 7), BPA (*n* = 7) and BPA/4T1 (*n* = 7) mice. The data shows the mean and standard deviation of the population percentage (* *p* < 0.05, ** *p* < 0.01 and *** *p* < 0.001).

Different immune populations were analysed in the spleen and PLN. As shown in Figure 4, at the splenic level, the administration of BPA to pregnant female mice produced a significant decrease of the T helper population in animals with scrotal 4T1-induced tumours (4C), without changes in total lymphocytes (4A) or T cytotoxic cells (4E). In the PLN, the antigenic tumour challenge caused a significant decrease in the total number of lymphocytes (4B), while treatment with BPA in the same animals, similarly to the spleen, caused a significant decrease in the helper T lymphocytes (4D) and no significant differences in the T cytotoxic population (4F).

**Figure 5:**
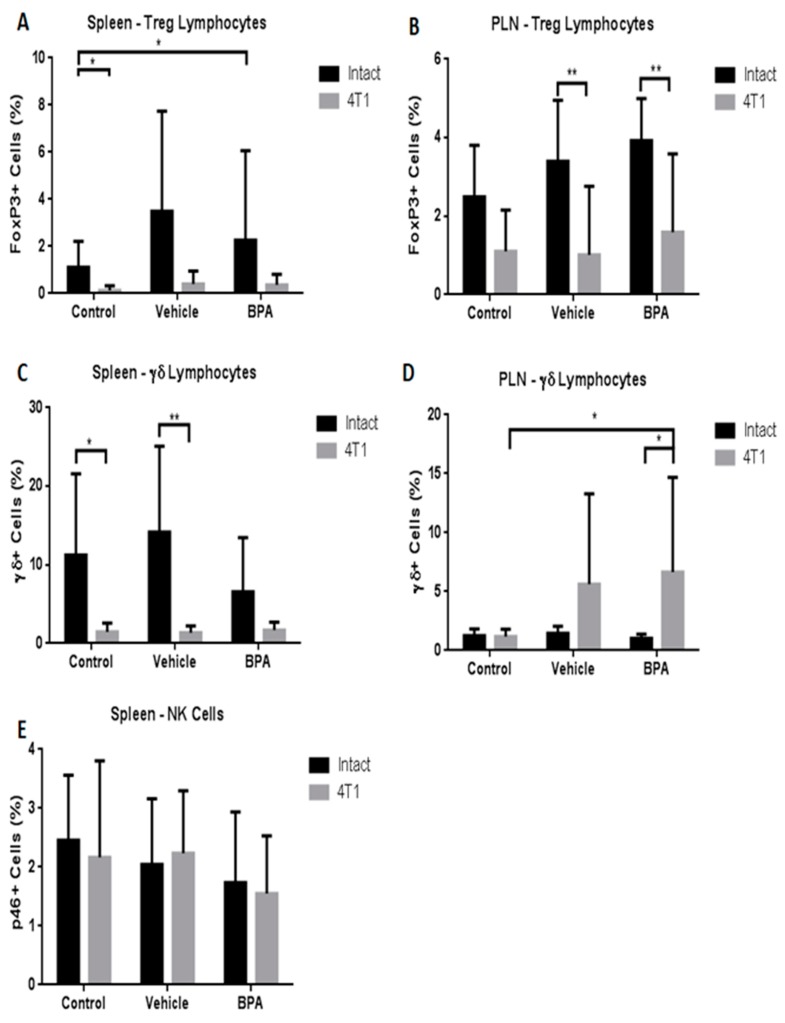
Percentages of regulatory T cells, T γδ cells (γδ+ cells) and natural killer (NK) subpopulations in the spleen and PLN in the offspring of BPA treated mice. Differences in cellular percentages by treatment in spleen and peripheral lymph nodes (PLN) in regulatory T cells (**A**,**B**), Tγδ lymphocytes (**C**,**D**), and NK (**E**) cells, control (*n* = 7), vehicle (*n* = 6), vehicle/4T1 (*n* = 7), BPA (*n* = 7), and BPA/4T1 (*n* = 5). The data shows the mean and standard deviation of the population percentage (* *p* < 0.05, ** *p* < 0.01 and *** *p* < 0.001).

In addition, we evaluated other immune cell populations in both immune organs. The results demonstrated that the Treg population was significantly diminished only in PLN by BPA action (Figure 5A,B). With regard to Tγδ cells, similar to total lymphocytes described above, the percentage of this population was negatively and positively regulated in the spleen (5C) and in PLN (5D), respectively, by the tumour challenge. Similar to T cytotoxic cells, no differences were observed in natural killer cells (NK) cell percentage in the spleen due to BPA exposure (5E).

**Figure 6:**
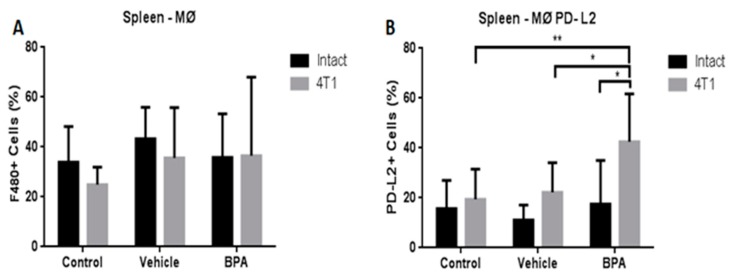
Percentages of total macrophages (MØ) and PD-L2+ macrophages (MØ-PD-L2) in the offspring of BPA treated animals. Macrophages (**A**), PD-L2 macrophages (**B**). Control (*n* = 7), vehicle (*n* = 6), vehicle/4T1 (*n* = 7), BPA (*n* = 7) and BPA/4T1 (*n* = 5). Data are expressed as the mean and standard deviation of the population percentage. * *p* < 0.05, ** *p* < 0.01.

Therefore, we also decided to study the modulation of the innate immune system represented by macrophages (MØ). Importantly, we evaluated the expression of programmed death-ligand 2 in these cells (MØ-PD-L2), since this protein regulates T cell activation and peripheral tolerance. Figure 6 shows that the percentage of macrophages in the spleen of the scrotal cancer model offspring of BPA treated mice was not altered by the exposure to the EDC (6A). However, the expression of the PD-L2 protein in these cells from animals with tumours that were exposed to BPA showed a significant increase compared with the vehicle and control groups (6B).

**Figure 7:**
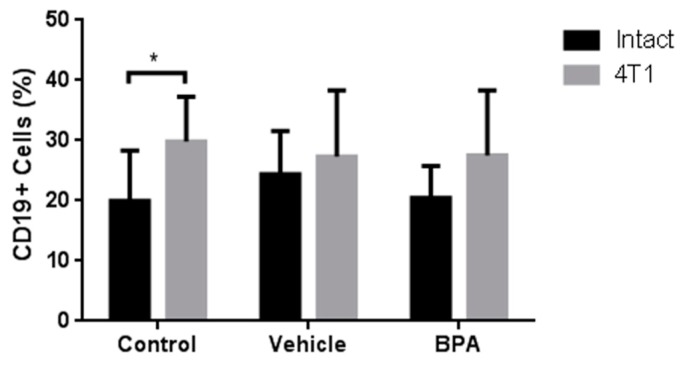
Percentage of B cells in the PLN of the offspring of BPA treated mice. PLN B Lymphocytes. Control (*n* = 7), vehicle (*n* = 6), vehicle/4T1 (*n* = 7), BPA (*n* = 7), and BPA / 4T1 (*n* = 5). Data are expressed as the mean and standard deviation of the population percentage. * *p* < 0.05.

As an integral immune view, we also decided to study the percentage of B lymphocytes (CD19+ cells), corresponding to the immune humoral response. As shown in Figure 7, BPA treatment has no significant effect on the B lymphocyte population in the spleen of animals with tumours (Figure 7) compared with the non-treated groups. What it is important to mention, is that the induction of tumours increased the levels of B cells, compared with the intact animals (Figure 7). In the case of PLN, there are no detectable levels of B cells in any group (not shown).

Additionally, all different immune cell populations analysed in both lymph organs above were also analysed in the tumour, and no significant differences were found between the different treatments (data not shown).

### 3.5. Relative Expression of Intra-Tumoural Cytokines

Next, we decided to evaluate the intra-tumoural expression of different cytokines related to the inflammatory and metastatic processes. As is shown in Table 2, BPA exposure caused a significant increase of anti-inflammatory cytokines, such as IL-4 and IL-10, compared to the control or vehicle groups. Additionally, a similarly significant effect was observed in the case of the pro-metastatic cytokine TGF-β. Interestingly, in the case of TNF-α and IFN-γ, there was a decrease in the intra-testicular tumour expression of both cytokines.

## 4. Discussion

In the last decades, the incidence of testicular cancer has increased. However, to date, little is known about the aetiology of testicular cancer, and much less, the immune control of it. The use of animal models in cancer experiments provide valuable information about the causes, development and the response of these diseases. Nevertheless, current animal models are far from simulating the testicular tumour process due to their genetic characteristics, for instance, the use of nude mice where the immune response is inhibited [18]. Another animal model previously used, the 129/Sv strain, shares histological characteristics present in humans, but only for paediatric tumours [19]. To our knowledge, this is the first study in which a testicular-like tumour model has been developed in a non-deficient mouse model. This represents an extremely useful strategy since the antitumoural immune response can be studied.

The present project evaluated whether BPA exposure during the perinatal stage might induce alterations of the immune response against tumour development. To do this, male adult mice, derived from pregnant female mice exposed to BPA, were inoculated in the scrotum with the 4T1 cancer cell line to develop testicular-like tumours, and the associated-immune response was evaluated. Although this tumour cell line is derived from breast tissue, the cells were administered into the reproductive tract of the male mice, then tumour cells successfully developed into tumours in a different site from its origin—allowing for the evaluation of the immune response at a systemic and tumoural level.

Our results clearly depict that BPA exposure favoured the growth of tumours derived in the mice scrotum, this notion is in accordance with other reports that have evaluated the effect of this compound in different types of cancer models [20,21]. Moreover, to evaluate whether BPA exposure could modify the structure and shape of the tumour cells, H&E stain analyses were performed. We observed that control groups and animals derived from BPA exposure presented similar tumour cell structures, with necrotic areas, as well as many hyperchromatic cell nuclei, which are common features of tumour tissues [22]. In other words, maternal exposure to BPA did not produce significant differences in the distribution and structure of the offspring tumour cell epithelium compared to the control groups. Although this compound has been shown to alter the morphology of hyperplastic epithelial cells derived from the rat mammary gland, it is important to mention that the administration period was different in other models [23].

Conversely, to determine whether BPA could affect the immune response during tumour development, the percentages of different immune cells were analysed in the spleen, PLN and tumours using flow cytometry. The results showed that in the mice with tumours, the percentage of the total lymphocyte population was lower in the spleen and lymph nodes, and BPA exposure did not result in any significant changes. The decrease in the total lymphocyte population seems to be due to the antigenic tumour challenge, which is associated with an underprivileged anti-tumour response [24]. This deficient immune response could be related to the significant decrease in helper T cells caused by exposure to BPA. Moreover, in the spleen of the animals with tumours, the number of regulatory T and Tγδ lymphocytes was decreased compared with the animals without tumours. In contrast, both immunoregulatory populations were significantly increased in PLN by BPA exposure. Interestingly, we did not find any of these populations in the tumour. It is known that regulatory T and Tγδ lymphocytes are considered as key players in immune tolerance as well as suppression of antitumour responses, specifically, Treg cells are associated with aggressive tumour phenotypes, and they home in on the tumours through the action of various cytokines [25]. Perhaps in our tumour model, the increase in Treg cells in the PLN is an intermediate step for their arrival to the tumour site.

Moreover, the PD-L2 marker is an important molecule related to the alternative phenotype of macrophages, and with the suppression of T lymphocyte proliferation [26]. Interestingly, we observed a significant increase in the percentage of PD-L2 on macrophages derived from mice exposed to BPA compared to the control groups, suggesting that in the testicular-like cancer model, macrophages expressing the PD-L2 marker govern an essential suppressive role of T cell proliferation and a pro-tumoural activity. In this work, we also observed a decrease of B lymphocytes in the spleen of animals, with tumours, that were exposed to BPA, is an important limiting factor for the decrease of their capacity to remove tumour cells. Nevertheless, deeper studies about the immune regulation by BPA are needed [27].

Many reports have mentioned that immune cellular and molecular components, such as cytokines, have multiple roles. In fact, cytokines are considered as meaningful components of the hallmarks of cancer [28]. In this sense, we evaluated the effect of BPA exposure on the expression of different intra-tumoural cytokines implicated in metastatic and pro or anti-inflammatory processes. The results showed that both anti-inflammatory cytokines, such as IL-4 and IL-10, were up-regulated in tumours derived from animals that had previous exposure with the EDC, while the expression of the pro-inflammatory counterpart (TNF-α and IFN-γ) was diminished. These results appear to contravene other reports where perinatal BPA exposure induces the secretion of pro-inflammatory mediators in the bone marrow-derived mast cells of 6-month-old adult mice offspring [29]. However, we did not study the BPA effect at a systemic level in the secretion of cytokines, but rather at the tumour level, indicating that the cytokine levels may be different depending on the tissue or cell type under investigation. Correlating the importance of macrophages in our model, and the secretion of TNF-α, it was reported that macrophages originated from mice exposed orally to BPA, and activated with LPS, decreased the TNF-α secretion [30], which is an important discovery since it is a key component in tumour cell clearance, thus exposure to BPA prevents tumour elimination.

This work is an initial exploration of how the immune response may be affected by an endocrine disruptor at the perinatal stage. Considering that experiments carried out on exposure to BPA and cells of the immune system are few, as well as different in terms of the form of exposure—prenatal, neonatal or perinatal, or even ex vivo or in vitro—as well as exposure doses, it is necessary to continue experimenting with comparable variables.

Although it has been described that cells of the immune system express oestrogen receptors, oestrogenic compounds can modulate the activity of these receptors [31]. However, the following questions remain: (1) How does BPA work? and (2) what are the molecular and genomic factors involved in this process? BPA can affect the immune system at important stages of development.

For a better understanding of the effects of BPA, there are several points that should be analysed: (1) The effect of BPA in early or intermediate stages of the antitumoural immune response; (2) The quantification of molecules with negative costimulatory actions; (3) The quantification of factors associated with malignant and proliferative development like TGF-β and VEGF to analyse the invasive and proliferative capacity associated with exposure to perinatal BPA.

## 5. Conclusions

The present work described a murine experimental model as a novel alternative for the study of the antitumoural immune response in the reproductive tract. In addition, perinatal exposure to BPA modifies the size of the tumour, it affects cells of the immune system during their development, and it has a side effect altering the specific immune response when the individual is adult.

## Figures and Tables

**Table 1:** Oligonucleotide sequences used for RT-PCR.

Oligonucleotide	Sequence	MT (°C)	Product (bp)
IL-4 Sense	TCATGGGATGATGATGATAACCTGCT	62	502
IL-4 Antisense	CCCATACTTTAGGAAGACACGGATT		
IL-10 Sense	AACTGGTAGAAGTGATGCCCCAGGCA	63	237
IL-10 Antisense	CTATGCAGTTGATGAAGATGTCAAA		
TNF-α Sense	GGCAGGTCTACTTTGGAGTCATTGC	63	300
TNF-α Antisense	ACATTCGAGGCTCCAGTGAATTCGG		
IFN-γ Sense	AGCGGCTGACTGAACTCAGATTGTAG	60	247
IFN-γ Antisense	GTCACAGTTTTCAGCTGTATAGGG		
TGF-β Sense	CTTCAGCTCCACAGAGAAGAACTGA	61	298
TGF-β Antisense	CACAATCATGTTGGACAACTGCTCC		
18S Sense	CGCGGTTCTATTTTGTTGGT	60	219
18S Antisense	AGTCGGCATCGTTTATGGTC		

Primers were designed based on sequenced mouse genes from the Gene Databank (NCBI, NIH).

**Table 2:** Cytokine pattern in testicular tumours of control, vehicle and BPA-treated mice.

Cytokine	Control	Vehicle	BPA
IL-4	20.5 ± 3.8	22.9 ± 6.3	** 83.2 ± 4.1
IL-10	10.1 ± 2.2	13.3 ± 1.2	** 57.0 ± 2.2
TNF-α	80.5 ± 8.3	78.9 ± 5.9	* 41.1 ± 8.2
IFN-γ	72.3 ± 3.8	69.4 ± 9.1	* 33.2 ± 9.1
TGF-β	39.5 ± 7.8	42.9 ± 8.4	* 89.6 ± 10.1

Relative expression [Optical Density (OD) cytokine/OD control gene (18s)]. Data are presented as the relative expression ± standard deviation. * *p* < 0.05, ** *p* < 0.01.
